# Clinicopathological characterization of *SMAD4*-mutated intestinal adenocarcinomas: A case-control study

**DOI:** 10.1371/journal.pone.0212142

**Published:** 2019-02-07

**Authors:** Xiaoyan Liao, Yansheng Hao, Xiaofei Zhang, Stephen Ward, Jane Houldsworth, Alexandros D. Polydorides, Noam Harpaz

**Affiliations:** 1 Department of Pathology, Icahn School of Medicine at Mount Sinai, New York, New York, United States of America; 2 Department of Pathology and Laboratory Medicine, University of Rochester Medical Center, Rochester, New York, United States of America; Universita degli Studi di Verona, ITALY

## Abstract

The *SMAD4* tumor suppressor gene product inhibits transforming growth factor-β-mediated signaling and is mutated in ~10% of colorectal carcinomas. The prognostic significance of *SMAD4* mutations has been controversial. We studied the pathological and clinical characteristics of *SMAD4*-mutated intestinal adenocarcinomas using a retrospective case-control study design. Cases and controls were identified among 443 primary adenocarcinomas that had undergone next generation DNA sequencing (NGS) with the Ion AmpliSeq Cancer Hotspot Panel v2, which evaluates 50 cancer-related genes. Twenty-eight *SMAD4*-mutated (*SMAD4*m) patients were matched 1:2 with 56 consecutive *SMAD4* wild-type (*SMAD4*wt) control patients from the same analysis stream. Compared with the *SMAD4*wt controls, the *SMAD4*m tumors were of higher stage (*P =* 0.026) and were more likely to feature mucinous differentiation (*P =* 0.0000), to occur in the setting of Crohn’s disease (*P =* 0.0041), and to harbor concurrent *RAS* mutations (*P =* 0.0178). Tumor mucin content was significantly correlated with mutations involving the MH2 domain of the *SMAD4* protein (*P =* 0.0338). Correspondence between mutation sites and morphology was demonstrated directly in a mixed adenocarcinoma and neuroendocrine tumor where *SMAD4* mutations involving different protein domains were found in histologically disparate tumor regions despite both containing identical *KRAS* and *TP53* mutations.

## Introduction

The transforming growth factor (TGF)-β signaling pathway is an important regulator of cellular and molecular processes in development and disease [1]. Among its downstream effectors, the *SMAD4* tumor suppressor gene product is important in intestinal carcinogenesis. Germline mutations in *SMAD4* cause juvenile polyposis syndrome (JPS) with an autosomal dominantly inherited predisposition to multiple gastrointestinal polyps and cancer [2]. *SMAD4* mutations have recently been reported in 5–20% sporadic colorectal carcinomas (CRC) where they were associated with distant metastases and/or poor prognosis in some studies but not others [3–7]. Missense mutations in the MH2 domain were the most common alterations. *SMAD4* mutations have also been observed in cancers with mucinous differentiation, especially those of high grade [8–11]. We carried out a retrospective case-control study aimed at characterizing the distinctive clinicopathological features of *SMAD4*-mutated intestinal adenocarcinomas (ACAs).

## Materials and methods

### Study population

We identified all primary ACAs of the large and small intestine (excluding the appendix) that underwent surgical resection and next generation sequencing (NGS) at our institution between 2013 and 2017. Information regarding the patients’ age, sex, family history, and any prior diagnosis of IBD were obtained from the electronic medical records. Patients that underwent neoadjuvant therapy before genetic analysis were excluded. For each *SMAD4*m tumor, the subsequent two *SMAD4*wt specimens in the analysis stream which contained other mutations were selected as controls.

Participant consent for this study was waived by the Institutional Review Board (IRB) of the Icahn School of Medicine at Mount Sinai.

### Histology and immunohistochemistry

Tumor grading and classification were assigned according to the WHO 2010 criteria [12]. Immunohistochemical stains were performed on a Dako Omnis or Ventana Ultra instrument. All antibodies were purchased as prediluted or optimized reagents, including Chromogranin (1:200, Dako, Santa Clara, CA), and SMAD4 (1:400, Abcam, Cambridge, MA). Mismatch repair status was determined by immunohistochemical staining for expression of MLH1, PMS2, MSH2 and MSH6 (pre-diluted, Dako).

### Next generation sequencing

Genomic DNA extraction was performed on paraffin-embedded tissue sections using the H&E-stained section as a guide and a cutoff of 60% tumor cellularity. DNA was amplified by multiplex PCR of targeted sequences in 50 genes using the Ion AmpliSeq Cancer Hotspot Panel v2 to generate an amplicon library. The genes included in this panel were *ABL1*, *AKT1*, *ALK*, *APC*, *ATM*, *BRAF*, *CDH1*, *CDKN2A*, *CSF1R*, *CTNNB1*, *EGFR*, *ERBB2*, *ERBB4*, *EZH2*, *FBXW7*, *FGFR1*, *FGFR2*, *FGFR3*, *FLT3*, *GNA11*,*GNAS*,*GNAQ*, *HNF1A*, *HRAS*, *IDH1*, *IDH2*, *JAK2*, *JAK3*, *KDR*, *KIT*, *KRAS*, *MET*, *MLH1*,*MPL*, *NOTCH1*, *NPM1*, *NRAS*, *PDGFRA*, *PIK3CA*, *PTEN*, *PTPN11*, *RB1*, *RET*, *SMAD4*, *SMARCB1*, *SMO*, *SRC*, *STK11*, *TP53*, *VHL*. The library was then clonally amplified by emulsion PCR, enriched and sequenced using the Ion AmpliSeq Cancer Hotspot Panel (v2, Thermo Fisher). The detection limit of this assay is 5% mutant alleles in a background of wild-type alleles. Reported variants from early cases were re-confirmed not to represent germline variants.

### Statistical analysis

Chi-square or Fisher’s exact test was applied with statistical significance defined as P<0.05. All analyses were performed using SAS version 9.2 (SAS Institute, Cary, NC, USA).

## Results

Of 443 primary intestinal ACAs (6 small bowel and 437 colorectal) that underwent sequencing, 28 (6.3%) harbored *SMAD4* mutations (*SMAD4*m). Based on the entire cohort, *SMAD4* mutations were significantly more prevalent among patients with Crohn’s disease than others (4/7 [57%] vs. 24/436 [5.5%], *P =* 0.0041; 3/5 [60%] vs. 24/436 [5.5%], P<0.0001 for CRCs only). The *SMAD4*m ACAs were then matched to ACAs with no mutations in *SMAD4* (*SMAD4*wt) from 56 patients, serving as controls. There were no significant differences between case and control groups with respect to patient’s age, gender or tumor location (Table 1). The proportion of ACAs with nodal metastases were significantly higher among cases compared to the controls (74% vs. 46%, *P =* 0.036, respectively). In addition, *SMAD4*m cases were significantly more likely to present at a higher overall TNM stage compared to controls (*P =* 0.026). Further review showed a higher proportion of tumor deposits in adipose tissue (9/19 [47%] vs. 12/56 [21%], *P* = 0.0296), and a higher percentage of lymph node metastasis (97/389 [25%] vs. 119/1167 [10%], P<0.0001) in cases than controls.

**Table 1:** Clinicopathological characteristics of *SMAD4*m cases and *SMAD4*wt controls.

		*SMAD4*m	*SMAD4*wt	*P*_value
**Patients (N)**		**28**	**56**	
**Median age (range)**		63 (38–83)	64 (34–85)	NS
**Sex**	Male	17 (61%)	26 (46%)	NS
	Female	11 (39%)	30 (54%)	
**Cases (N)**		**28**	**57**^**#**^	
**Tumor site**	Terminal ileum/ICV	2 (7%)	2 (4%)	NS
	Cecum/Ascending colon	10 (36%)	21 (37%)	NS
	Transverse colon	2 (7%)	10 (18%)	NS
	Descending colon	1 (4%)	1 (2%)	NS
	Rectosigmoid	8 (29%)	23 (40%)	NS
	NOS	5 (18%)*	0	NS
**Cases (N)**		22*	**56**	
**Nodal metastasis**	None	6 (27%)	30 (54%)	**0.036**
	Present	16 (73%)	26 (46%)	
**TNM stage**	Stage I	2 (9%)	7 (13%)	**0.026**
	Stage II	4 (18%)	23 (41%)	
	Stage III	10 (45%)	23 (41%)	
	Stage IV	6 (27%)	3 (5%)	

*Six *SMAD4*m tumors were metastatic with a diagnosis of colorectal cancer based on a combination of histopathology findings, clinical and imaging data.

^#^One control patient had two synchronous tumors, one from cecum, and one from transverse colon, which were staged according to the highest.

Compared to *SMAD4*wt controls, *SMAD4*m ACAs were significantly more likely to be classified as mucinous (>50% mucin content, 17/28 [68%] vs. 9/58 [14%], P<0.00001; Table 2) or as having mucinous features (>5% mucin content, 9/28 [32%] vs. 4/54 [7%], *P =* 0.0022). Importantly, this association correlated with the protein domain harboring the mutation, where 10 of 12 (83%) *SMAD4*m ACAs that carried mutations in the MH2 domain had mucinous features (>5% mucin content), compared with 7 of 16 (44%) *SMAD4*m ACAs having mucinous features when the mutation involved other *SMAD4* domains (*P =* .0338).

**Table 2:** *SMAD4* mutations and mucinous differentiation.

		*SMAD4*m (N = 28)		*SMAD4*wt (N = 57)	P_value
**Mucinous**	Yes	17 (68%)		9 (16%)	**<0.00001**
**differentiation**	No/unknown	11 (32%)		48 (84%)	
		**MH2 domain (N = 12)**	**Other domains (N = 16)**		
**Mucinous**	Yes	10 (83%)	7 (44%)		**0.0338**
**differentiation**	No/unknown	2 (17%)	9 (56%)		

In all cases, *SMAD4* mutations were accompanied by mutations in other genes (Table 3, S1 Table). The most frequent were *RAS* mutations, i.e., *KRAS* (n = 20) and *NRAS* (n = 2). Cumulatively, *RAS* mutations occurred at a higher rate in SMADm cases than in the *SMAD4*wt control group (79% vs 52%, *P =* 0.0178). Nevertheless, mucinous differentiation in *SMAD4*m cases occurred independently of *KRAS* mutation status, i.e. SAMD4m/*RAS* wild-type tumors and *SMAD4*m/*RAS* mutated tumors have similar frequency of mucinous features (2/5 [40%] vs. 15/23 [65%], *P =* 0.583). Other recurrent mutations involving *TP53*, *APC*, *PIK3CA*, and *BRAF* were less common and occurred at similar rates between the two groups. Rare mutations in *FBXW7*, *PTEN*, *ATM*, and *CTNNB1* were also detected, but were too few for statistical comparison. A slightly higher proportion of *SMAD4*m than *SMAD4*wt tumors were MMR proficient (20/22 [91%] vs. 40/54 [74%]); however, the difference did not reach statistical significance (*P =* 0.103).

**Table 3:** Molecular characteristics of *SMAD4*m tumors.

		*SMAD4*m	*SMAD4*wt	P_value
**Cases (N)**		**28**	56*	
**Genetic mutations**	KRAS/NRAS	22 (79%)	29 (52%)	**0.0178**
	TP53	13(46%)	26 (46%)	NS
	APC	11(39%)	18 (32%)	NS
	PIK3CA	3 (11%)	14 (25%)	NS
	BRAF	2 (7%)	11 (20%)	NS
	FBXW7	2 (7%)	5 (9%)	NS
	PTEN	1 (4%)	4 (7%)	NS
	ATM	1 (4%)	3 (5%)	NS
	CTNNB1	0	3 (5%)	NS
**Cases tested (N)**		**22**	**54**	
**MMR by IHC**	MSS	20 (91%)	40 (74%)	**0.103**
	MSI-H	2 (9%)	14 (26%)	

* The control patient with two synchronous tumors had only transverse colon tumor sequenced.

**Fig 1:**
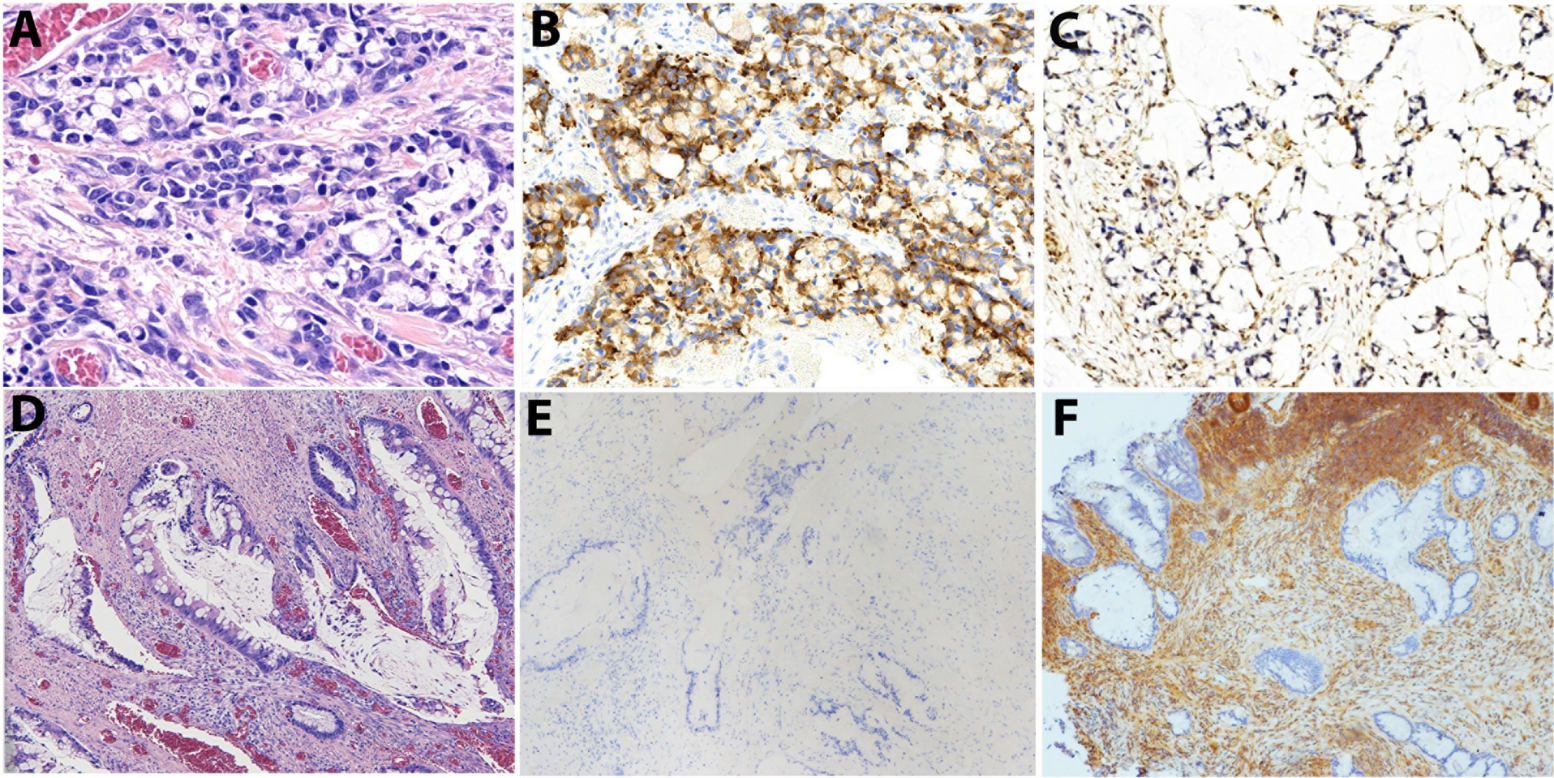
A case of mucinous ACA of the ascending colon with two distinct but contiguous phenotypes: crypt cell/neuroendocrine carcinoma (A-C) and classical mucinous ACA (D-F), Immunohistochemical stains confirmed expression of Chromogranin in only the crypt cell/neuroendocrine component (B, E) and loss of SMAD4 expression in both regions of the tumor (C, F). Magnification: 200x.

Correspondence between the site of *SMAD4* mutation and tumor morphology was demonstrated directly in a case of mixed adenocarcinoma and neuroendocrine tumor (Case #20, S1 Table). In this particular case, contiguous but histologically disparate regions of the tumor comprising crypt cell neuroendocrine carcinoma (a.k.a. goblet cell carcinoid) and classical mucinous adenocarcinoma (Fig 1**)** harbored distinct *SMAD4* mutations, MH2 domain (c.1082G>A) mutation and c.379T>A in the latter, respectively, despite harboring identical mutations of *KRAS* (c.35G>T) and *TP53* (c.742C>T). The results suggest divergent differentiation from a single clone.

## Discussion

The protein products of the *SMAD* genes are essential mediators of the TGF-β signaling pathway, playing critical roles in growth inhibition of normal epithelial cells. Dysregulation of this pathway leads to carcinogenesis, and *SMAD4* dysfunction is the most frequent cause. Earlier studies exploring the relationship between SMAD4 protein and carcinogenesis assayed loss of SMAD4 protein expression by immunohistochemical staining, which however may or may not be due to *SMAD4* genetic mutations [5]. Table 4 summarizes the 10 studies that investigated the implications of *SMAD4* genetic mutations in intestinal ACAs. As shown, the prognostic significance of *SMAD4* mutations in intestinal ACAs was first reported in 1999 using the PCR method, yet the association between *SMAD4* gene mutations and mucinous morphology was not described until 2013 [8], particularly in high-grade vs. low-grade mucinous ACAs [9,10]. A retrospective study of 90 *SMAD4*-mutated ACAs reported poorer survival rates in patients with *SMAD4*-mutated tumors, but did not include mucinous morphology as a potential risk factor [3]. Similarly, Mizuno et al reported worse survival, but did not report tumor morphology of the *SMAD4*-mutated cancers [6], while Khan et al reported association of mucinous morphology with *SMAD4* mutation and worse prognosis [11]. Recognizing these knowledge gaps, our comprehensive study using a stringent retrospective case-control design confirms that *SMAD4* mutations are associated with higher tumor stage, nodal metastasis, tumor deposits in adipose tissue, mucinous morphology, and *RAS* mutations.

**Table 4:** Summary of published studies investigating *SMAD4* mutations in intestinal ACAs.

Author, Year of publication	# of *SMAD4*-mutated ACAs	% of ACAs tested	Testing method	Prognostic significance	Correlation with other genes	Morphological correlation
Miyaki, 1999 [4]	21	11.9%	PCR- SSCP	Distant metastasis	Not done	Not done
Alazzouzi, 2005 [5]	5	6.25%	PCR	Not associated with survival	allelic imbalance in chromosome 18q21	Not done
Fleming, 2012 [8]	64	8.6%	Single-nucleotide polymorphism microarray analysis	No relationship to AJCC stage, T stage, N stage, or lymphovascular invasion	Not done	Mucinous morphology
Yoshioka, 2015 [9]	7	20%	Ion AmpliSeq Cancer Hotspot Panel	Not done	Not done	High-grade mucinous morphology
Goswami, 2015 [7]	Not known	Not known	Next-generation sequencing hotspot mutation panel	Distant metastasis	Not done	Not done
Chang, 2016 [10]	9	8.3%	MassARRAY-based mutation detection methods	Not done	Not done	Mucinous morphology
Mehrvarz Sarshekeh, 2017 [3]	90	12.2%	HiSeq sequencing system hotspot testing	Associated with shorter overall survival; but not age, stage at presentation, colonic location, distant metastasis, or tumor grade	Not done	Not done
Mizuno, 2018 [6]	37	13%	Next-generation 50-gene sequencing platform	Worse survival	RAS	Not done
Khan 2018 [11]	226	12.3%	Ion Torrent AmpliSeq Cancer Panel Primers	Not done	Not done	Mucinous morphology
Liao, 2018	28	5.6%	Next-generation 50-gene sequencing platform	Higher tumor stage, nodal and distant metastasis	RAS	Mucinous morphology

Among all *SMAD4* hotspot mutations, the MH2 domain is the most important, frequently containing missense mutations including Asp351 (D351), Pro356 (P356) and Arg361 (R361) which result in loss of function, and Ala406 (A406), Lys428 (K428), and Arg515 within the L3 loop which compromise *SMAD4* binding to SMAD2/3 [8,13,14]. We found that *SMAD4* mutations, particularly those involving the MH2 domain and abrogating protein function, are highly correlated with mucinous morphology. Further support for a correlation between tumor morphology and *SMAD4* mutational status was obtained from a rare mixed adenocarcinoma and neuroendocrine carcinoma of the colon, in which histologically divergent tumor regions manifested distinct *SMAD4* mutations despite conservation of identical *KRAS* and *TP53* mutations.

In agreement with previous studies [4,7], we found that *SMAD4*m tumors were significantly more likely to have tumor deposits, nodal metastases and higher stage than corresponding *SMAD4*wt tumors. The mechanism is unknown but a study of *in vitro* CRC cell lines has implicated the effects of *SMAD4* expression on tumor microenvironment [15]. Correlation between *SMAD4* status and tumor stage has been described in other organs. For example, *SMAD4* mutations are not typical of pancreatic intraductal papillary mucinous neoplasms but occur in up to 16% of invasive carcinomas that are associated with IPMN [16]. Likewise, low grade appendiceal mucinous neoplasms (LAMN) do not usually harbor *SMAD4* mutations until there is intraperitoneal spread [17].

In this study, we also investigated the relationship between *SMAD4* and other gene mutations, especially *RAS* genes since these two are closely associated. *KRAS* mutations have been reported to correlate with mucinous differentiation in CRCs [11,18], yet the mucinous differentiation in *SMAD4*m tumors is independent of *KRAS* mutation status. Tumors with *RAS* mutations are known to be intrinsically resistant to anti-EGFR therapy [19,20]; however, it has been shown that *SMAD4* mutation is an independent factor of resistance to anti-EGFR therapy [3]. Indeed, SMAD4 inactivation also predicted worse survival in patient receiving fluorouracil-based therapy [21]. We did not find associations between *SMAD4* and *PTEN* mutations, although a recent study showed that concurrent loss of SMAD4 and PTEN protein expression may lead to worse outcomes in patients with CRC [22]. In addition, a trend for *SMAD4* mutation to associate with MMR proficiency is noted but not proved in this study, likely due to small case numbers.

Prior studies of ACA complicating Crohn’s disease either did not report or did not observe increased proportions of *SMAD4* mutations compared to sporadic ACAs [23,24]. Nevertheless, the potential role of SMAD4 function in Crohn’s disease was demonstrated in a recent study showing downregulation of the SMAD4 protein in ileal epithelial cells of patients with Crohn’s disease [25]. We found a significantly high percentage of *SMAD4* mutations in ACAs from patients with Crohn’s disease, with or without inclusion of small intestine ACAs, warranting future larger studies to validate and further explore this association.

In conclusion, we present a comprehensive clinicopathological and molecular characterization of *SMAD4*-mutated intestinal ACAs, using case-control methodology. We identified an association of *SMAD4* mutations with mucinous morphology, advanced tumor stage, concomitant RAS mutations and divergent differentiation in a rare mixed adenocarcinoma and neuroendocrine carcinoma.

## Supporting information

S1 Table Raw genetics data from for cases (n = 28) and controls (n = 56). (XLSX)
